# Effects of wearable devices on gait, balance, and motor function in people with Parkinson’s disease: a systematic review and meta-analysis

**DOI:** 10.3389/fpubh.2026.1846005

**Published:** 2026-07-15

**Authors:** Qin Zhong, Duanyong Liu, Yiyong Xu, Gui Xiao

**Affiliations:** School of Nursing, Jiangxi University of Chinese Medicine, Nanchang, Jiangxi, China

**Keywords:** balance, gait, meta-analysis, motor function, Parkinson’s disease, wearable devices

## Abstract

**Objective:**

This study aimed to evaluate the effects of wearable devices on gait, balance, and motor function in people with Parkinson’s Disease (PwPD) through a systematic review and meta-analysis of randomized controlled trials (RCTs).

**Methods:**

A comprehensive literature search was conducted across international and Chinese databases to identify relevant studies published from database inception through December 2025. Data were independently screened, extracted, and analyzed using RevMan 5.4 and Stata 16. Subgroup analyses were conducted based on the FITT principle, including intervention duration, weekly frequency, feedback modality, and device category.

**Results:**

A total of 13 RCTs comprising 380 participants were included. The meta-analysis revealed that wearable devices were associated with selective improvements in certain gait and motor outcomes among PwPD. Specifically, improvements were observed in several key measures, including walking speed [MD = 0.07, 95% CI (0.01, 0.12), *p* = 0.02], TUGT [MD = −2.00, 95% CI (−3.57, −0.43), *p* = 0.01], and UPDRS-III [SMD = −0.33, 95% CI (−0.59, −0.06), *p* = 0.02]. No significant improvements were observed for stride length, step length, step cadence, FOGQ, BBS, Mini-BESTest, DS time, 6MWT, or PDQ scores. Subgroup analyses suggested that interventions lasting 7–12 weeks and those performed 3–5 times per week may be associated with improvements in walking speed in PwPD, while no significant effects were observed for shorter duration or other frequency categories. Subgroup analyses by device category and feedback modality did not show statistically significant differences in walking speed.

**Conclusion:**

These findings suggest that wearable devices may selectively improve gait (walking speed), dynamic balance (TUGT), and motor function (UPDRS-III) in PwPD, but evidence for and quality of life remains limited. Further high-quality randomized controlled trials with standardized protocols are warranted to confirm these findings and guide clinical implementation.

**Systematic review registration:**

https://www.crd.york.ac.uk/PROSPERO/view/CRD420251246681, identifier, CRD420251246681.

## 1 Introduction

Parkinson’s disease (PD) is the fastest-growing neurodegenerative disorder worldwide, with its global prevalence having doubled over the past 25 years to more than 8.5 million cases (1). As a progressive neurological disorder, PD is characterized by cardinal motor symptoms including gait disturbances, postural instability, and bradykinesia. These impairments are the primary contributors to an elevated risk of falls, progressive functional decline, and a substantial deterioration in quality of life (2, 3). The resulting disability not only imposes a profound personal burden on individuals living with PD but also translates into a considerable public health challenge, driven by rising direct healthcare expenditures, indirect costs related to productivity loss, and the physical and emotional strain placed on caregivers and support systems (4, 5). Consequently, developing and implementing effective strategies for managing motor dysfunction represents one of the most urgent priorities in contemporary PwPD care and long-term rehabilitation.

Conventional physiotherapy (e.g., deep brain stimulation) and pharmacotherapy (e.g., dopamine replacement) form the cornerstone of managing motor symptoms in PD, demonstrating established efficacy in improving overall function (6, 7). However, sustaining long-term improvements in gait, balance, and motor function, which are critical for quality of life, remains challenging. These challenges are compounded by the progressive nature of PD, where symptom severity and manifestation often fluctuate over time. Conventional approaches often require specialist supervision, limiting their accessibility and frequency in real-world settings (8). Additionally, as PD progresses, treatment regimens typically demand repeated adjustments to address symptom evolution and side effects (9, 10). This creates a need for more flexible, adaptive, and patient-centered solutions. These considerations motivate the exploration of adjunctive, technology-supported strategies that can extend therapeutic reach.

Wearable devices, ranging from inertial measurement units (IMUs) and pressure-sensitive insoles to smart textiles and exoskeletons, represent a promising complementary approach that is being increasingly integrated into PwPD rehabilitation (11, 12). The proposed mechanisms through which these devices improve motor function are multifaceted. They leverage real-time, multi-modal sensory feedback—such as auditory, visual, or haptic cues—to compensate for impaired proprioception and facilitate movement initiation and coordination (13). Additionally, by enabling closed-loop, adaptive stimulation or cueing, wearables can promote neuroplasticity through repetitive, task-specific training, thereby reinforcing motor learning and enhancing neural circuit efficiency (14). Critically, wearables facilitate ecologically valid, home-based exercise to overcome spatial and temporal access barriers, and their data-driven nature supports adaptive, personalized intervention dosing, which is essential for managing progressive symptoms (15, 16). Thus, they represent a paradigm shift towards accessible, responsive, and data-informed motor rehabilitation.

Research on the application of wearable devices in PwPD rehabilitation has grown substantially. Numerous studies have demonstrated their potential benefits across multiple motor domains: for example, sensor-based cueing has been shown to improve stride length and gait speed (17); real-time biofeedback has enhanced postural stability (18); and integrated training systems have facilitated overall motor coordination (19). However, significant heterogeneity exists across these studies, particularly in terms of sample size, intervention protocols, and device types. Moreover, the optimal training parameters—such as session duration, frequency—remain poorly defined, resulting in a lack of strong, consistent experimental evidence to guide clinical implementation. While several qualitative reviews have synthesized this expanding literature (20, 21), existing systematic reviews and meta-analyses often adopt a narrow scope, mainly focusing on gait and balance function while paying limited attention to overall motor function, and fail to provide clear guidance on the optimal wearable device intervention plan (such as intervention duration and frequency) (22, 23).

This selective focus restricts the ability to draw definitive conclusions regarding the comprehensive efficacy of wearable technologies. Consequently, there is a clear need for a systematic review and meta-analysis that quantitatively integrates evidence across all key motor domains to establish robust, clinically meaningful conclusions.

In this study, we implemented an extensive meta-analysis to investigate the impact of wearable devices as a promising complementary treatment on the gait, balance, and motor function of PwPD. By synthesizing fragmented evidence and reconciling inconsistent findings, this research aims to advance the field and provide new insights and robust intervention strategies for the rehabilitation therapy for motor dysfunction of PwPD.

## 2 Materials and methods

This systematic review and meta-analysis adheres to the Preferred Reporting Items for Systematic Reviews and Meta-Analyses (PRISMA) reporting guidelines. The protocol was registered in PROSPERO (CRD420251246681).

### 2.1 Inclusion and exclusion criteria

Inclusion criteria were as follows: (1) participants (P): patients diagnosed with Parkinson’s disease, no restrictions based on sex, nationality, ethnicity, or stage of the Hoehn and Yahr scale. (2) Intervention (I): received wearable devices training. (3) Control (C): adopted either training without wearable devices or no training. (4) Outcome (O): included the assessment of gait capacity, balance ability and motor function, evaluated using the walking speed, stride length, step length, step cadence, Freezing of Gait Questionnaire (FOGQ), Berg Balance Scale (BBS), Mini-Balance Evaluation Systems Test (Mini- BESTest), Timed Up and Go (TUG) test, Double Support time (DS time), Unified Parkinson’s Disease Rating Scale, Part III (UPDRS-III), Six-Minute Walk Test (6MWT) and Parkinson’s Disease Questionnaire (PDQ). (5) Study Design (S): randomized controlled trials.

Exclusion criteria were as follows: (1) full texts could not be obtained or duplicate literature; (2) studies with incomplete data that could not be extracted for the calculation of mean values and standard deviations; (3) the judgement indicators of the research results were not clearly defined, data were missing or ambiguous, or the results could not be converted and merged; (4) the outcome measures of the RCTs were not included among our defined outcomes.

### 2.2 Search strategy

Two researchers (both participated in training on Evidence-based Nursing, Qin Zhong and Gui Xiao) independently searched eight databases, including CNKI, Chongqing VIP, SinoMed, Wanfang, PubMed, EMbase, Cochrane Library, and Web of Science, for randomized controlled trial (RCT) exploring the effects of wearable devices on PwPD up to December 2025. At the same time, the literature was traced in combination with the “snowball” method. The search strategy combined Medical Subject Headings (MeSH) terms and free-text keywords. The Chinese search terms were: (帕金森) 和 (可穿戴 或 传感器 或 追踪器 或 加速度计). The English search terms were: (Parkinson Disease OR PD OR Parkinsonism OR Paralysis Agitans) AND (Wearable Device OR Accelerometer OR Gyroscope OR sensor OR Shoe OR Insole OR Activity Tracker) AND (Clinical Trials as Topic OR Clinical Trial OR Random Allocation OR Randomized Controlled Trial OR Placebo OR RCT). The search terms were adjusted according to the specific requirements of each database. For example, in PubMed, the detailed search strategy is presented in Table 1. Complete search strategies for each database are provided in Supplementary material 1.

**Table 1:** PubMed search strategy.

Serial number	Search strategy
#1	(“Parkinson Disease”[Mesh]) OR (((((((((((Idiopathic Parkinson Disease[Title/Abstract]) OR (Idiopathic Parkinson’s Disease[Title/Abstract])) OR (Lewy Body Parkinson Disease[Title/Abstract])) OR (Lewy Body Parkinson’s Disease[Title/Abstract])) OR (Paralysis Agitans[Title/Abstract])) OR (Parkinson Disease, Idiopathic[Title/Abstract])) OR (Parkinson’s Disease[Title/Abstract])) OR (Parkinson’s Disease, Idiopathic[Title/ Abstract])) OR (Parkinson’s Disease, Lewy Body[Title/Abstract])) OR (Primary Parkinsonism[Title/Abstract])) OR (Parkinsonism, Primary[Title/Abstract]))
#2	(((((((((“Wearable Electronic Devices”[Mesh]) OR ((((((((((((((Device, Wearable Electronic[Title/Abstract]) OR (Electronic Device, Wearable[Title/Abstract])) OR (Wearable Electronic Device[Title/Abstract])) OR (Wearable Devices[Title/Abstract])) OR (Device, Wearable[Title/Abstract])) OR (Wearable Device[Title/Abstract])) OR (Wearable Technology[Title/Abstract])) OR (Technology, Wearable[Title/Abstract])) OR (Wearable Technologies[Title/Abstract])) OR (Electronic Skin[Title/Abstract])) OR (Skin, Electronic[Title/Abstract])) OR (Wearable Computer[Title/Abstract])) OR (Computer, Wearable[Title/Abstract])) OR (Wearable Computers[Title/Abstract]))) OR (Motion Capture Devices[Title/Abstract])) OR (Inertial Measurement Unit[Title/Abstract])) OR (Acceleromet*[Title/Abstract])) OR (gyroscope*[Title/ Abstract])) OR (sensor*[Title/Abstract])) OR (shoe*[Title/Abstract])) OR (Insole*[Title/ Abstract])) OR (activity tracker*[Title/Abstract])
#3	((((“Clinical Trial” [Publication Type]) OR “Clinical Trials as Topic”[Mesh]) OR “Random Allocation”[Mesh]) OR “Randomized Controlled Trial” [Publication Type]) OR ((((random*[Title/Abstract]) OR (Clinical Trial[Title/Abstract])) OR (placebo[Title/ Abstract])) OR (RCT[Title/Abstract]))
#4	#1 AND #2 AND #3

### 2.3 Study selection and data extraction

The studies were exported using the EndNote reference manager (Version X9, Clarivate Analytics, Philadelphia, PA, USA). Two researchers (Qin Zhong and Gui Xiao) independently examined all the studies. After removing duplicates according to predefined criteria, they independently screened and excluded studies that did not meet the inclusion criteria. The following information was extracted from the remaining studies using a standardized form: (1) characteristics of the study (first author, publication year, country); (2) characteristics of participant (sample size, age, disease duration, Hoehn & Yahr stage); (3) intervention protocol (types of wearable cueing device, feedback characteristics, experimental and control interventions, frequency and duration of training); (4) outcome measures (gait capacity, balance ability, motor function outcomes). Any discrepancies or divergences in data extraction were resolved through discussion between the two reviewers to reach consensus, with a third reviewer (Duanyong Liu) making the final decision when agreement could not be achieved.

### 2.4 Quality assessment

Two researchers (Qin Zhong and Gui Xiao) independently assessed the risk of bias for all included studies using the Cochrane Risk of Bias tool (from the Cochrane Handbook for Systematic Reviews of Interventions, version 5.3.0) (24). The assessment covered random sequence generation, allocation concealment, blinding of participants and personnel, blinding of outcome assessment, and incomplete outcome data, selective reporting, and other biases. Each item was judged as having a low, high, or uncertain risk of bias. In cases where discrepancies arose between the two researchers, a third reviewer (Duanyong Liu) was consulted for discussion or adjudication until a consensus was reached.

### 2.5 Data analysis

The analysis and exhibition of the survey results in this study were executed using Manager 5.4 and Stata 64 software. The gait capacity, balance ability, motor function outcomes examined are continuous variables, analyzed using weighted mean difference (WMD) and 95% confidence interval (CI). Heterogeneity was subsequently assessed using Cochran’s Q test and the I^2^ statistic. For outcomes with more than three studies, a fixed-effects model applied when heterogeneity was low (*p* > 0.10 and *I^2^* < 50%) and a random-effects model employed otherwise (25). For outcomes with ≤3 studies, a random-effects model was applied by default, regardless of the heterogeneity test results. Subgroup analyses were then performed based on wearable devices intervention features (duration and frequency of training). Furthermore, *p* ≤ 0.05 indicates a significant difference, demonstrating statistical significance in the meta-analysis results. To address publication bias, both qualitative (funnel plot) and quantitative (Egger’s test) methods were used.

## 3 Results

### 3.1 Search selection

**Figure 1:**
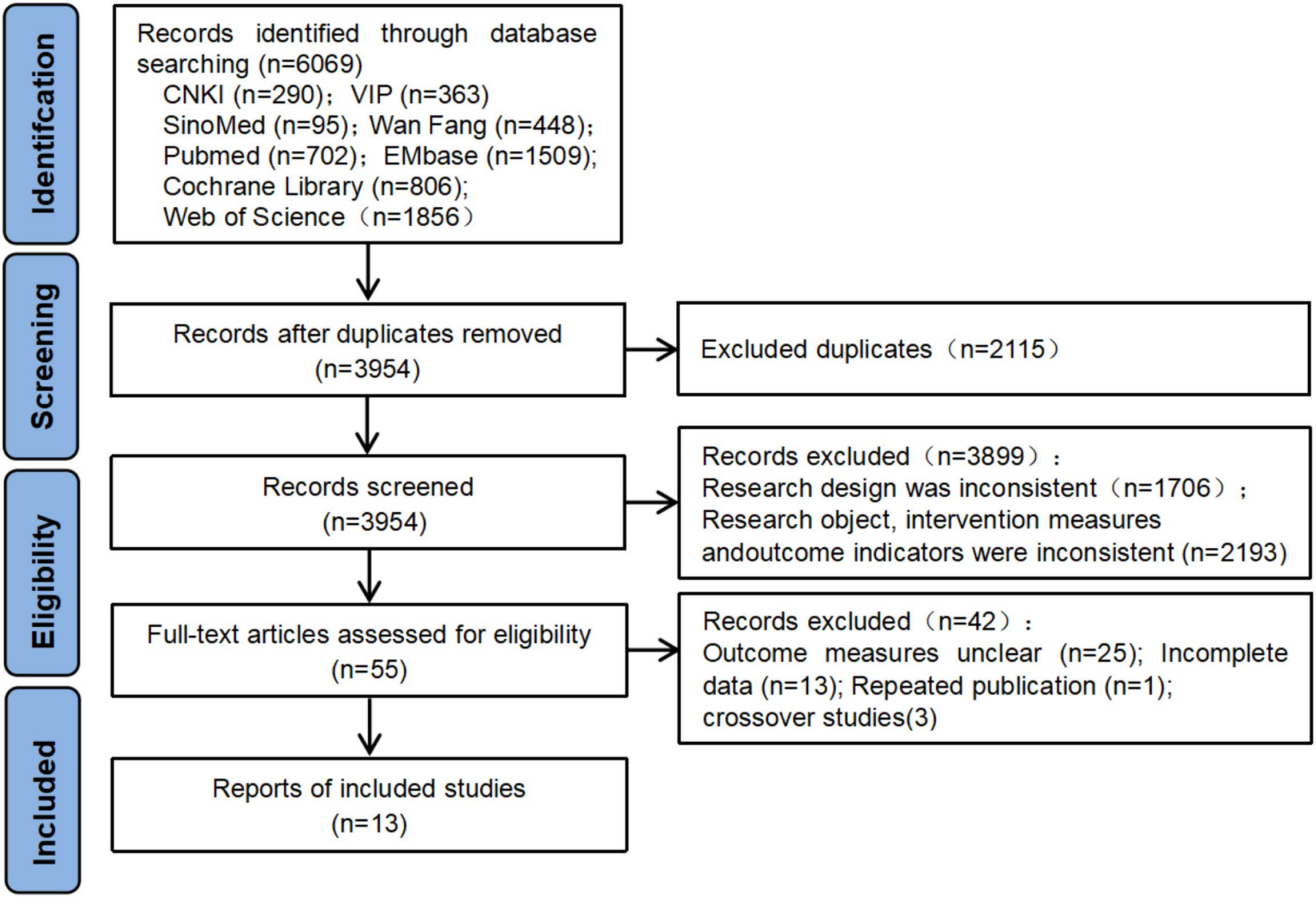
Literature search results and process.

Figure 1 details the search process for the studies. A comprehensive search of seven databases yielded 6,069 records. Following the removal of 2,115 duplicates, 3,954 studies were screened. After preliminary screening, 55 articles underwent full-text review, of which 13 studies (26–38) met the final inclusion criteria. 3 of the included interventional studies (39–41) were crossover trials. These studies were excluded from the main meta-analysis because crossover designs are not methodologically compatible with parallel-group designs in the context of this review. A separate exploratory analysis of these three studies is provided in the Supplementary materials.

### 3.2 Study characteristics

This study included 13 studies published between 2012 and 2024 (Table 2). A total of 380 participants were included, with numbers in each study ranging from 11 to 52. The studies were conducted in Germany, Italy, Canada, South Korea, Japan, Brazil and Belgium. Participants’ ages ranged from 60 to 76 years, with intervention durations ranging from 1 to 12 weeks, frequencies ranging from 1 to 7 times per week. The types of feedback provided by wearable cueing devices included auditory, proprioceptive, combined visual–auditory and vision-proprioception feedback.

**Table 2:** Characteristics of included studies.

First author (Year)	Country	Sample size	Age, mean (SD) (years)	Disease duration	Hoehn & Yahr stage	Wearable device	Feedback modality	Device category	Intervention		Duration/frequency	Outcomes
									Experimental group	Control group		
Mayo et al. (2024) (26)	Canada	T: 14 C: 7	T: 70.20 ± 8.50 C: 70.70 ± 8.80	N/A	2–3	Heel2Toe sensor	Auditory	Active sensor-based feedback	Training with Heel2Toe sensor	Training according to the manual	12 weeks 2 times/day	(11)(12)
Bartolo et al. (2024) (27)	Italy	T: 26 C: 26	T: 73.00 ± 7.3 C: 70.30 ± 11.0	T: 9.40 ± 3.1 C: 9.80 ± 3.9	2–4	Q-Walk	Vision	Active sensor-based feedback	New wearable visual cueing system (Q-Walk)	Traditional visual cues	2 weeks 5 sessions/week 10 sessions	(1)(3)(9) (10)
Pollet et al. (2023) (28)	Italy	T: 21 C: 21	T: 72.00 ± 7.05 C: 72.00 ± 5.02	N/A	1–4	PRO-STEP	Proprioception	Passive proprioceptive	Wear newly designed custom-made insole flPRO-STEP	Wear flat sham insole	10 weeks 6 h/day	(1)(2)(4) (6)(8)
Gryfe et al. (2022) (29)	Canada	T: 13 C: 14	T: 67.60 ± 5.90 C: 70.70 ± 7.30	N/A	1–4	Exoskeleton device	Proprioception	Exoskeleton wearable devices	Perform sessions whilst wearing the exoskeleton device	Perform exercise sessions in standard of care fashion	8 weeks 2 sessions/week 16 sessions	(1)(5)(7) (10)(11) (12)
Kim et al. (2022) (30)	South Korea	T: 22 C: 22	T: 68.70 ± 6.90 C: 67.50 ± 9.30	T: 111.80 ± 69.80 C: 104.60 ± 53.40 (month)	2.5–3	Walkbot-S	Vision, auditory (multimodal)	Exoskeleton wearable devices	Perform gait training using a treadmill- based exoskeleton robot	Perform gait training using a treadmill	4 weeks 3 sessions/week 12 sessions	(1)(5)(6) (8)(10)
Kawashima et al. (2022) (31)	Japan	T: 5 C: 7	T: 76.60 ± 5.30 C: 75.40 ± 5.70	T: 11.20 ± 5.80 C: 12.40 ± 4.60	2–4	SMA exoskeleton	Proprioception	Exoskeleton wearable devices	Gait training with SMA	Gait training without SMA	3 months 10 session 30 min/session	(1)(3)(5) (6)(10) (12)
Nakano et al. (2022) (32)	Japan	T: 15 C: 14	T: 71.13 ± 7.91 C: 71.36 ± 7.91	N/A	2–4	Insoles with a toe-grip bar	Proprioception	Passive proprioceptive	Wear shoes having insoles witha toe-grip bar	Wear shoes having insoles without a toe-grip bar	4 weeks 5 days/week	(1)(3)
Carpinella et al. (2017) (33)	Italy	T: 17 C: 20	T: 73.00 ± 7.10 C: 75.60 ± 8.20	T: 7.50 ± 3.20 C: 10.30 ± 5.70	2–4	Gamepad	Vision, Auditory (Multimodal)	Active sensor-based feedback	Biofeedback training with Gamepad	Structured physiotherapy without biofeedback	3 times/week 20 session 45 min	(1)(5)(6) (8)(10) (12)
Volpe et al. (2017) (34)	Italy	T: 10 C: 8	T: 69.18 ± 7.61 C: 63.37 ± 6.89	T: 7.82 ± 4.00 C: 8.12 ± 2.90	3	Sensory-motor orthotic	Proprioception	Passive proprioceptive	Balance training program with sensory-motor orthotic	Balance training program without sensory-motor orthotic	2 weeks 5 days/week 10 session 50 min	(6)(8)(10) (11)(12)
Lirani-Silva et al. (2017) (35)	Brazil	T: 10 C: 9	T: 70.40 ± 6.87 C: 72.00 ± 6.20	N/A	1–3	Textured insole	Proprioception	Passive proprioceptive	Textured insole	Conventional insole	1 week	(1)(2)(4) (9)
Chomiak et al. (2017) (36)	Canada	T: 5 C: 6	T: 70.8 ± 5.6 C: 69.0 ± 5.7	T: 15.4 ± 5.4 C: 11.2 ± 5.0	1–4	The Ambulosono platform; iPod Touch	Auditory	Active sensor-based feedback	Receive contingent music playback	Receive contingent CBC podcast	4 week 3 days/week 10–20 min/ day	(5)
Ginis et al. (2016) (37)	Belgium	T: 20 C: 18	T: 67.30 ± 8.13 C: 66.11 ± 8.07	T: 10.65 ± 5.39 C: 11.67 ± 7.63	2–3	CuPiD system	Auditory	Active sensor-based feedback	CuPiD, in which a smartphone application offered positive and corrective feedback on gait	Active control, in which personalized gait advice was provided	6 weeks 3 times/week 30 min	(1)(2)(5) (7)(9) (10)
EI-Tamawy et al. (2012) (38)	Egypt	T: 15 C: 15	T: 61.40 ± 7.28 C: 63.20 ± 5.60	T: 4.00 ± 0.90 C: 3.80 ± 0.90	2–3	The vibratory devices (VDs)	Proprioception	Passive proprioceptive	PNF and vibratory stimuli+ physiotherapy program	Physiotherapy program	8 weeks 3 times/week 30 min	(2)(4)

(1) Walking speed, (2)stride length, (3) step length, (4) step cadence, (5) FOGQ, (6) BBS, (7) Mini- BESTest (Brief BEST Test), (8) TUGT, (9) DS time, (10) UPDRS-III, (11) 6MWT, (12) PDQ. T, experimental group; C, control group; N/A, not applicable.

### 3.3 Methodological quality assessment

**Figure 2:**
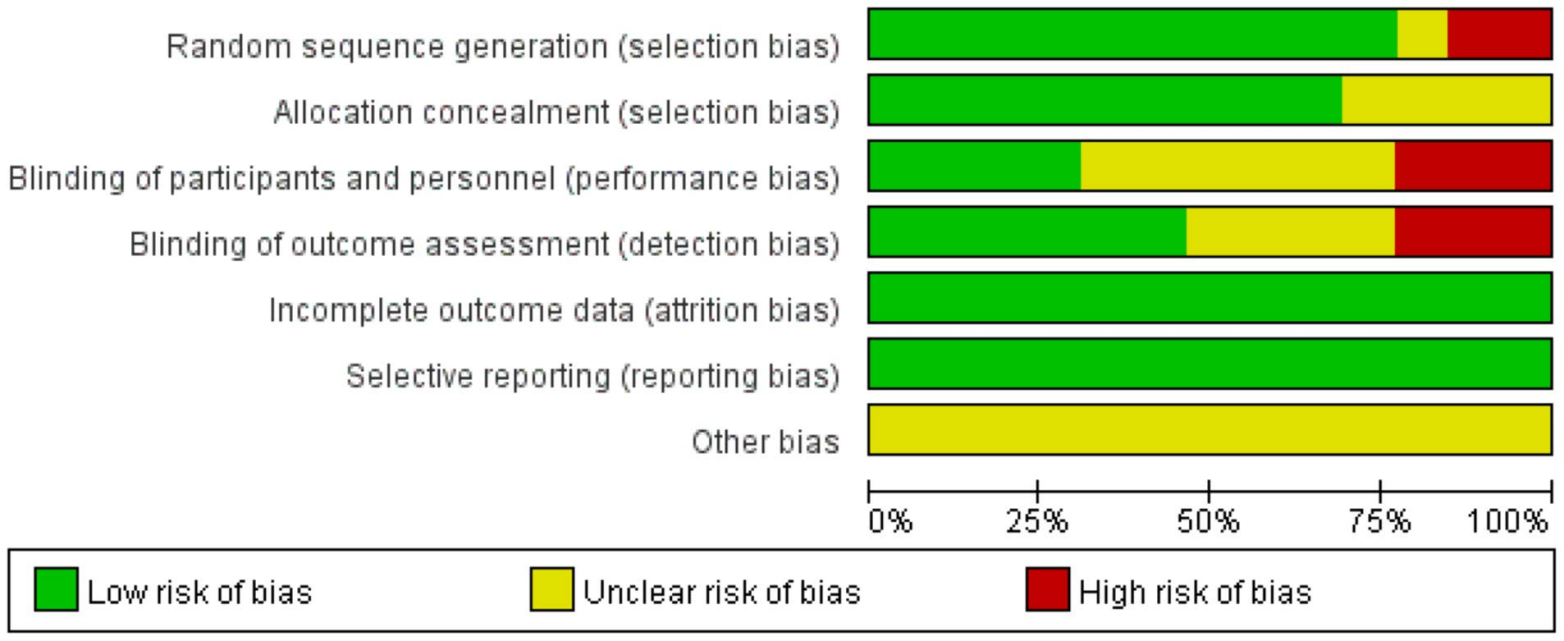
Risk of bias assessment.

**Figure 3:**
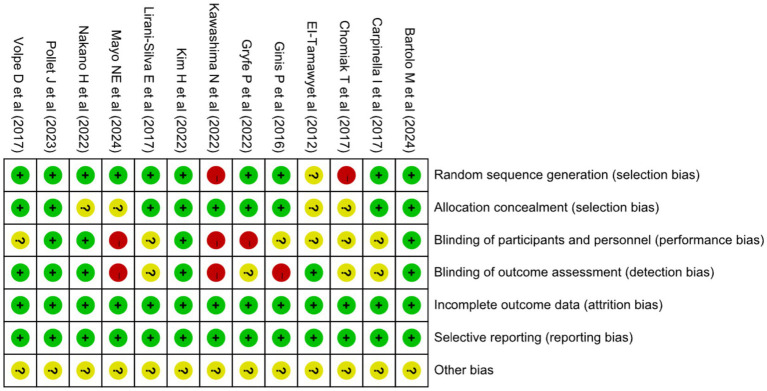
Summary of bias risk for each bias risk.

The methodological quality of the included studies was assessed using the Cochrane Risk of Bias Tool. 10 of them provided a detailed description of the methods used to generate the randomization sequence (26–30, 32–35, 37), 9 studies provided detailed information on allocation concealment (27–31, 33–35, 37), 4 studies reported on the implementation of blinding by participants and personnel (27, 28, 30, 32), 7 studies reported on the implementation of blinding by outcome assessment (27, 28, 30, 32, 34, 38). Data reporting was complete for all 13 studies, and any missing data or easons for missing data were thoroughly described. All 13 studies demonstrated selective outcome reporting. For the category of “other biases,” none of the studies provided details, and they were rated as having an unclear risk. The detailed results of the quality assessment are shown in Figures 2, 3.

### 3.4 Meta-analysis results

#### 3.4.1 Gait capacity

##### 3.4.1.1 Walking speed

**Figure 4:**
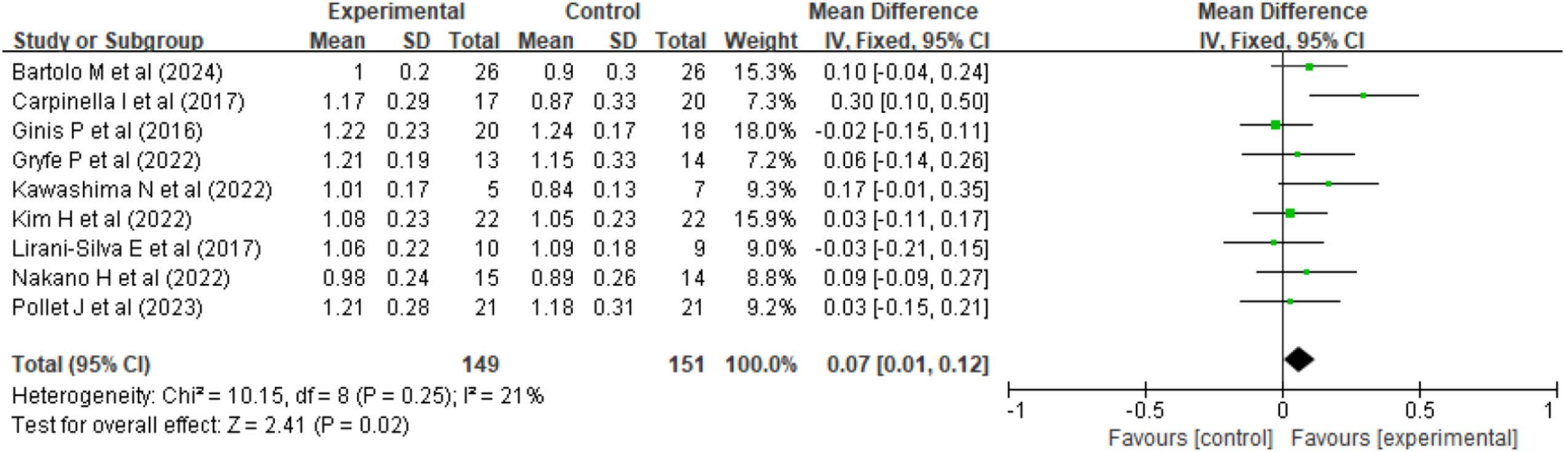
Forest plots for walking speed.

A total of nine studies (27–33, 37, 38) encompassing 300 patients reported walking speed and were included in the meta-analysis. The heterogeneity analysis showed no significant heterogeneity among the studies (*I^2^* = 21%, *p* = 0.25). Therefore, a fixed-effects model indicated that the walking speed in the experimental group was significantly higher than that in the control group [MD = 0.07, 95% CI (0.01, 0.12), *p* = 0.02], as illustrated in Figure 4. 3 cross experiments were added, and the results were not affected, as shown in Supplementary Figure 1.

**Figure 5:**
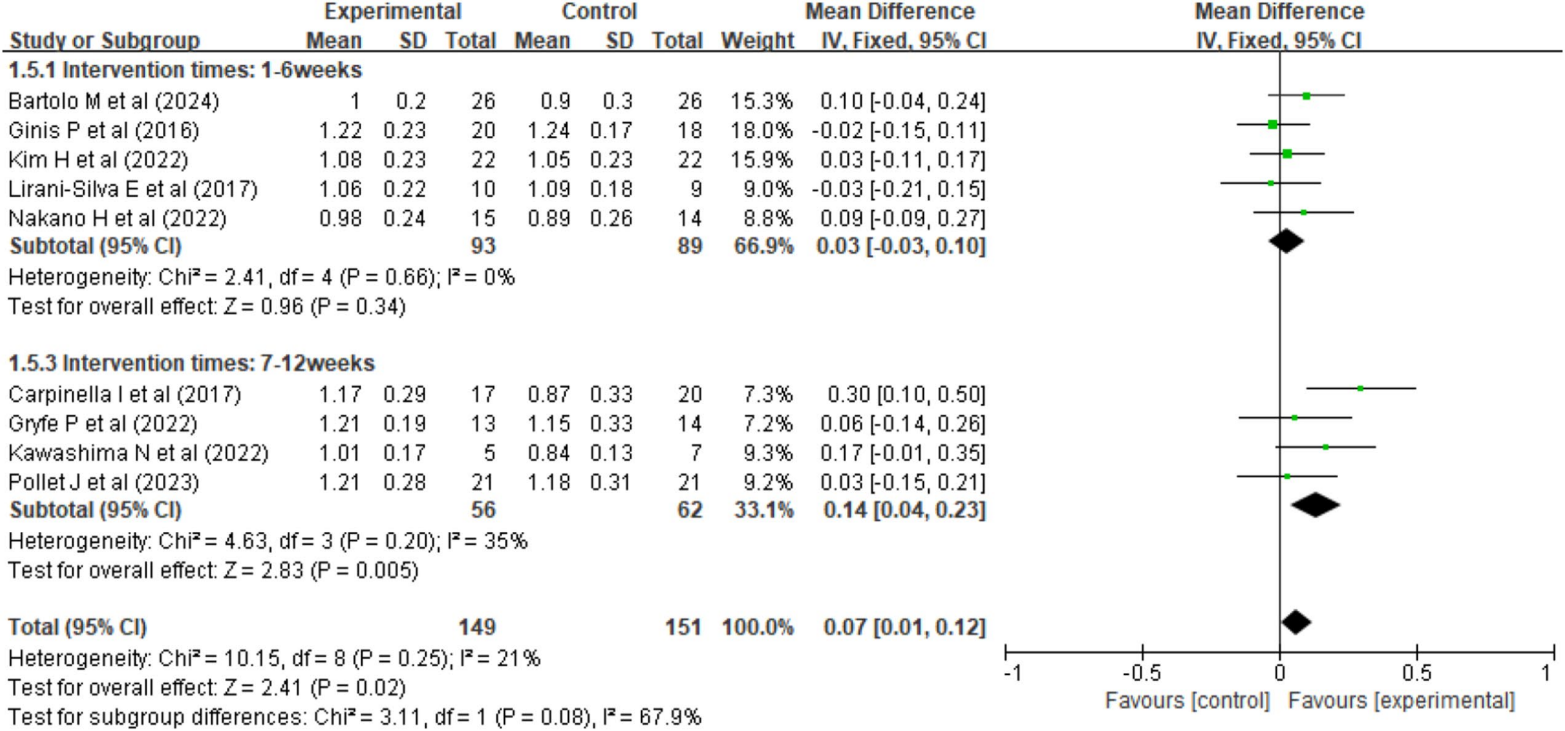
Subgroup analysis of the effect of wearable device on the walking speed in PwPD based on intervention duration.

A subgroup analysis was conducted based on the duration of wearable device intervention, dividing the studies into two subgroups: 1–6 weeks and 7–12 weeks. The results of the subgroup analysis revealed differences in the effects of wearable device on walking speed performance among PwPD based on intervention duration. In the 7–12 weeks group, the pooled effect size [MD = 0.14, 95% CI (0.04, 0.23), *p* = 0.01] indicated a statistically significant improvement in walking speed performance, demonstrating the effectiveness of wearable device in enhancing gait capacity in PwPD. However, in the 1–6 weeks group [MD = 0.03, 95% CI (−0.03, 0.10), *p* = 0.34], the results did not reach statistical significance. Heterogeneity analysis showed that the two groups exhibited low heterogeneity (*I^2^* = 0%, *I^2^* = 35%). Detailed results are presented in Figure 5.

**Figure 6:**
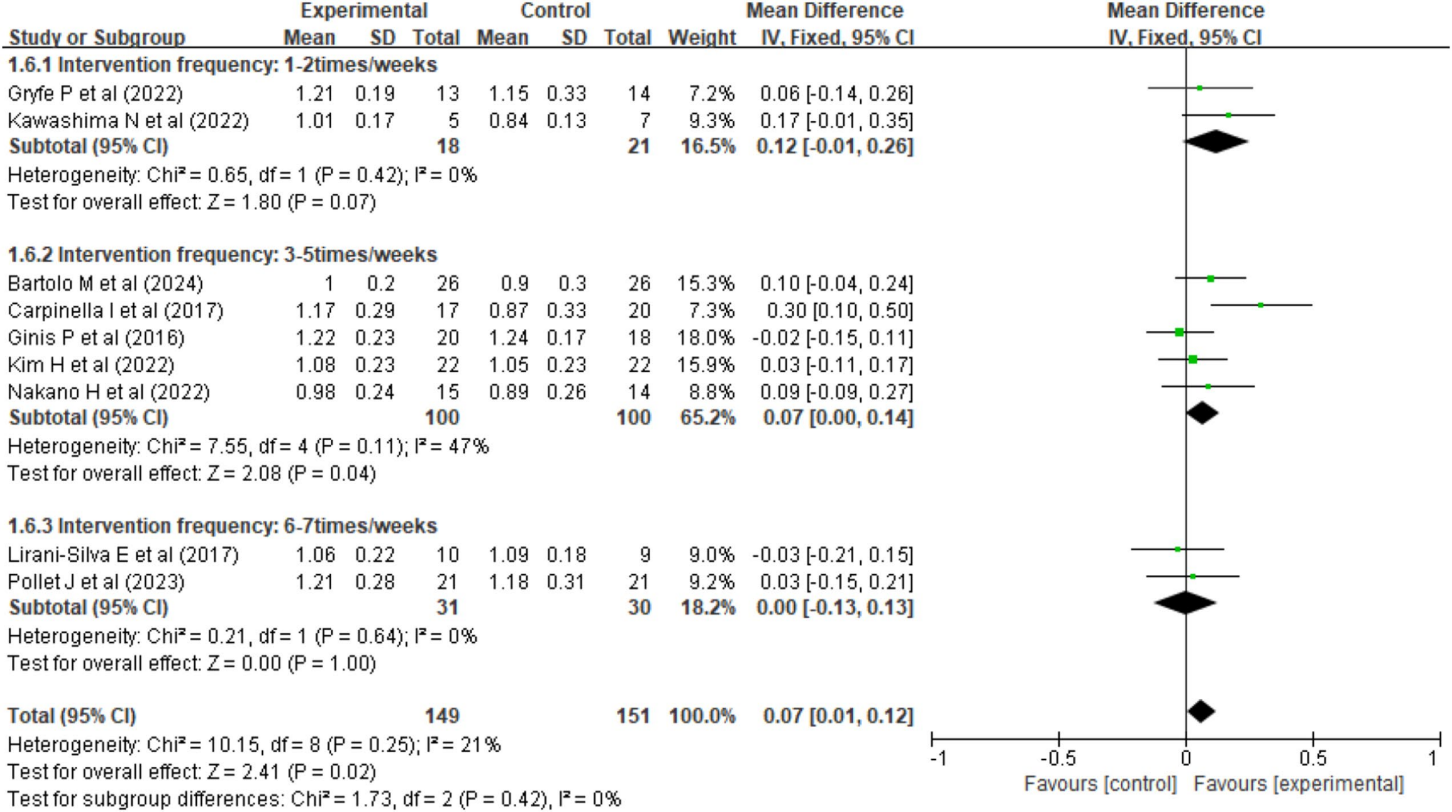
Subgroup analysis of the effect of wearable device on the walking speed in PwPD based on weekly intervention frequency.

The studies were categorized into two subgroups based on the frequency of wearable device intervention: 1–2 times/week, 3–5 times/week and 6–7 times/week. The results of the subgroup analysis revealed differences in the effects of wearable device on walking speed performance among PwPD based on the frequency of each intervention session. In the 3–5 times/week group, the pooled effect size [MD = 0.07, 95% CI (0.00, 0.14), *p* = 0.04] indicated a statistically significant improvement in walking speed performance, demonstrating the effectiveness of wearable device in enhancing gait capacity in PwPD. However, in the 1–2 times/week [MD = 0.12, 95% CI (−0.01, 0.26), *p* = 0.07] and 6–7 times/week [MD = 0.02, 95% CI (−0.13, 0.13), *p* = 1.00] group, the results did not reach statistical ignificance. Heterogeneity analysis showed that the three groups exhibited low heterogeneity (*I^2^* = 0%, *I^2^* = 47%, *I^2^* = 0%). Detailed results are presented in Figure 6.

The subgroup forest plots by feedback modality and device category are provided in Supplementary Figures 2, 3, respectively. Subgroup analysis by feedback modality showed no statistically significant difference across auditory, visual, proprioceptive, and multimodal feedback modalities, as shown in Supplementary Figure 2. Although most subgroup estimates favoured the experimental intervention, the confidence intervals were wide and crossed the line of no effect. Similarly, When studies were grouped by device category, no significant subgroup difference was observed (Supplementary Figure 3). These findings suggest that the effect of wearable devices on walking speed was not clearly modified by device category or feedback modality.

##### 3.4.1.2 Stride length

**Figure 7:**
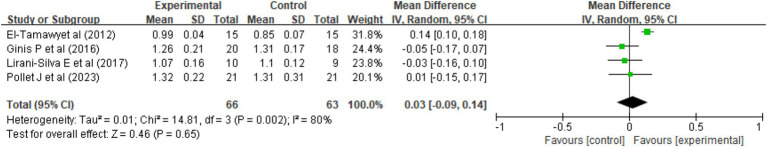
Forest plots for stride length.

A total of four studies (28, 35, 37, 38) encompassing 129 patients reported stride length and were included in the meta-analysis. The heterogeneity analysis showed no significant heterogeneity among the studies (*I^2^* = 80%, *p* = 0.002). A random-effects model demonstrated that no significant difference in stride length was detected between the groups [MD = 0.03, 95% CI (−0.09, 0.14), *p* = 0.65], as presented in Figure 7.

##### 3.4.1.3 Step length

**Figure 8:**
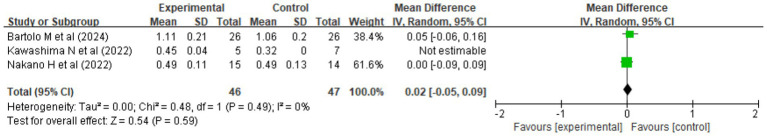
Forest plots for step length.

A total of three studies (27, 31, 32) encompassing 81 patients reported stride length and were included in the meta-analysis. The heterogeneity analysis showed no significant heterogeneity among the studies (*I^2^* = 0%, *p* = 0.49). A random-effects model demonstrated that no significant difference in stride length was detected between the groups [MD = 0.02, 95% CI (−0.05, 0.09), *p* = 0.59], as presented in Figure 8.

##### 3.4.1.4 Step cadence

**Figure 9:**
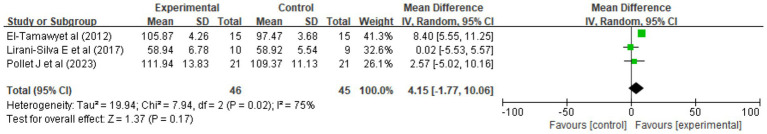
Forest plots for step cadence.

A total of 3 studies (28, 35, 38) encompassing 77 patients reported step cadence and were included in the meta-analysis. The heterogeneity analysis showed no significant heterogeneity among the studies (*I^2^* = 75%, *p* = 0.02). A random-effects model demonstrated that no significant difference in step cadence was detected between the groups [MD = 4.15, 95% CI (−1.77, 10.06), *p* = 0.02], as depicted in Figure 9.

##### 3.4.1.5 FOGQ

**Figure 10:**
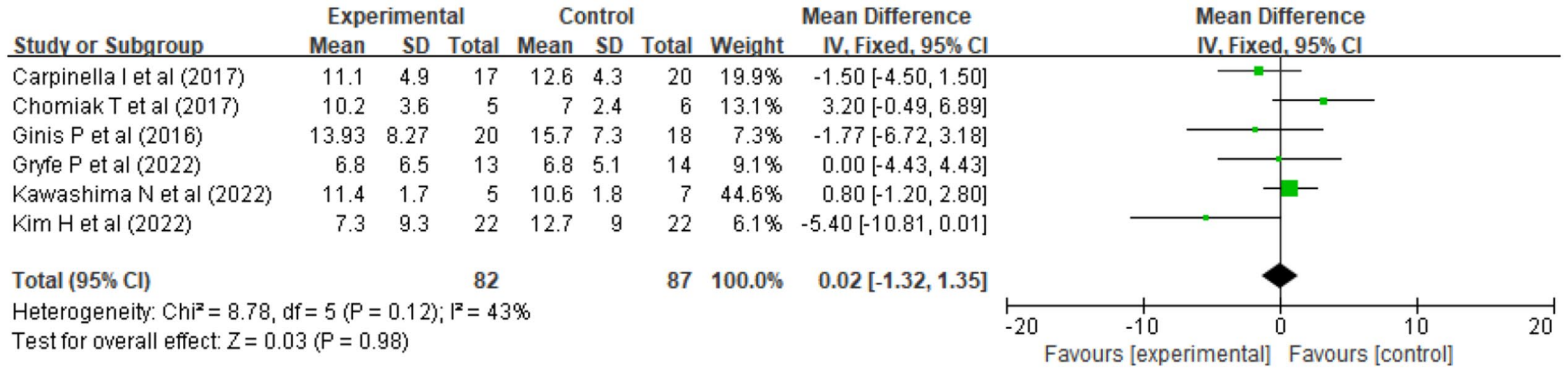
Forest plots for FOGQ.

Six studies (29–31, 33, 36, 37) involving 169 patients reported FOGQ scores and were included in the meta-analysis. The analysis revealed low heterogeneity (*I^2^* = 43%, *p* = 0.12). A fixed-effects model demonstrated that no significant difference in FOGQ was detected between the groups [MD = 0.02, 95% CI (−1.32, 1.35), *p* = 0.98], as presented in Figure 10.

#### 3.4.2 Balance ability

##### 3.4.2.1 BBS

**Figure 11:**
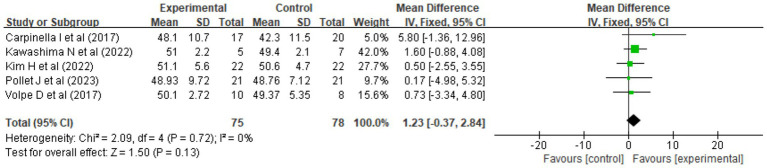
Forest plots for BBS.

Five studies (28, 30, 31, 33, 34) involving 153 patients reported the BBS and were included in the meta-analysis. The heterogeneity analysis showed no significant heterogeneity among the studies (*I^2^* = 0%, *p* = 0.72). A fixed-effects model indicated that no significant difference in BBS was detected between the groups [MD = 1.23, 95% CI (−0.37, 2.84), *p* = 0.13], as illustrated in Figure 11.

##### 3.4.2.2 Mini- BESTest

**Figure 12:**
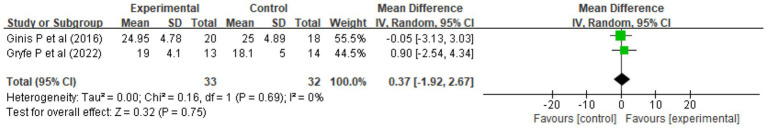
Forest plots for Mini- BESTest.

Two studies (29, 37) encompassing 65 patients reported Mini- BESTest and were included in the meta-analysis. The heterogeneity analysis showed no significant heterogeneity among the studies (*I^2^* = 0%, *p* = 0.69). A random-effects model demonstrated that no significant difference in Mini- BESTest was detected between the groups [MD = 0.37, 95% CI (−1.92, 2.67), *p* = 0.75], as presented in Figure 12.

##### 3.4.2.3 TUGT

**Figure 13:**
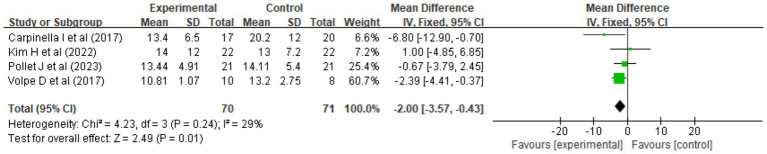
Forest plots for TUGT.

Four studies (28, 30, 33, 34) involving 141 patients reported the TUGT and were included in the meta-analysis. The heterogeneity analysis showed low heterogeneity among thestudies (*I^2^* = 29%, *p* = 0.24). Therefore, a fixed-effects model indicated that wearable device significantly improved TUGT levels in PwPD [MD = −2.00, 95% CI (−3.57, −0.43), *p* = 0.01], as illustrated in Figure 13.

##### 3.4.2.4 DS

**Figure 14:**
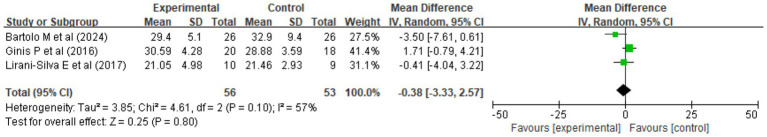
Forest plots for DS.

Three studies (27, 35, 37) encompassing 57 patients reported DS and were included in the meta-analysis. The heterogeneity analysis showed no significant heterogeneity among the studies (*I^2^* = 0%, *p* = 0.10). A random-effects model indicated that no significant difference in DS was detected between the groups [MD = −0.38, 95% CI (−3.33, 2.57), *p* = 0.80], as presented in Figure 14.

#### 3.4.3 Motor function

##### 3.4.3.1 UPDRS III

**Figure 15:**
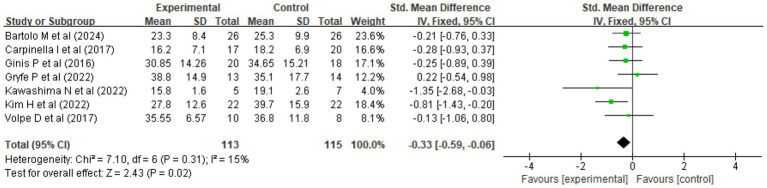
Forest plots for UPDRS III.

Seven studies (27, 29–31, 33, 34, 37) involving 228 patients reported the UPDRS III and were included in the meta-analysis. The heterogeneity analysis showed no significant heterogeneity among thestudies (*I^2^* = 15%, *p* = 0.31). Therefore, a fixed-effects model indicated that wearable device significantly improved UPDRS III levels in PwPD [SMD = −0.33, 95% CI (−0.59, −0.06), *p* = 0.02], as illustrated in Figure 15. Considering the differences between UPDRS-III and MDS-UPDRS-III, we conducted separate meta-analyses for each version. The results showed a statistically significant effect of wearable devices on UPDRS-III (*P* = < 0.008; Supplementary Figure 4), but no significant effect on MDS-UPDRS-III (*p* = 0.08; Supplementary Figure 5).

##### 3.4.3.2 6MWT

**Figure 16:**
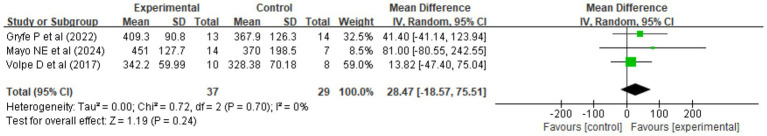
Forest plots for 6MWT.

Three studies (26, 29, 34) encompassing 66 patients reported 6MWT and were included in the meta-analysis. The heterogeneity analysis showed no significant heterogeneity among the studies (*I^2^* = 0%, *p* = 0.70). A random-effects model indicated that no significant difference in 6MWT was detected between the groups [MD = 28.47, 95% CI (−18.57, 75.51), *p* = 0.24], as presented in Figure 16.

#### 3.4.4 Quality of life

**Figure 17:**
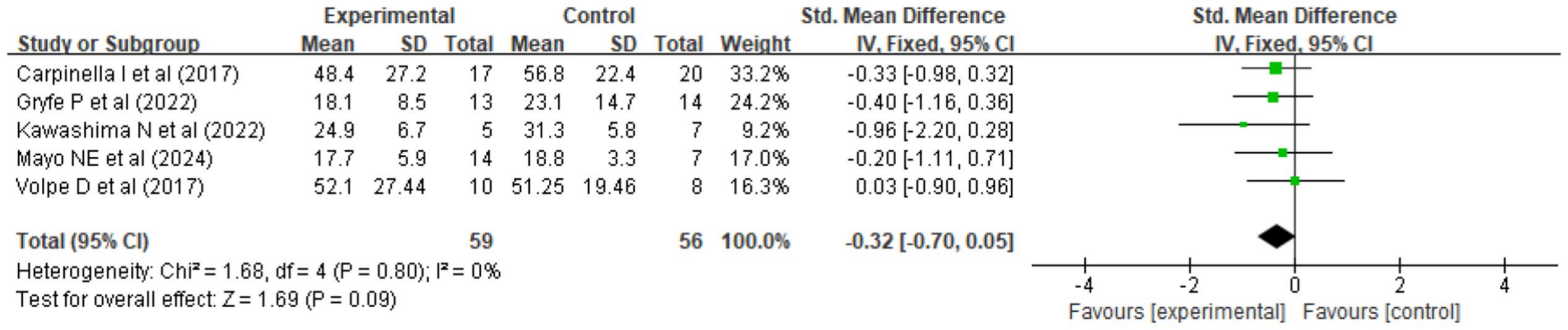
Forest plots for PDQ.

Five studies (26, 29, 31, 33, 34) assessed quality of life using PDQ instruments. Four studies used the PDQ-39 (30, 32, 36, 37), whereas 1 study (26) used the PDQ-8. Therefore, the primary meta-analysis pooled these outcomes using SMD. The heterogeneity analysis showed no significant heterogeneity among the studies (*I^2^* = 0%, *p* = 0.80). A fixed-effects model demonstrated that no significant difference in PDQ levels in PwPD [SMD = −0.32, 95% CI (−0.70, 0.05), *p* = 0.09], as presented in Figure 17.

### 3.5 Publication bias

**Figure 18:**
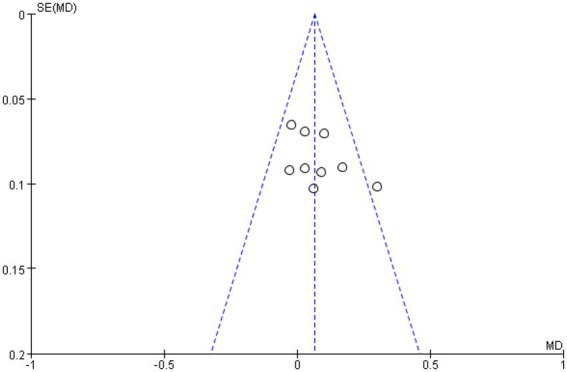
Funnel plot of all included studies for walking speed.

**Figure 19:**
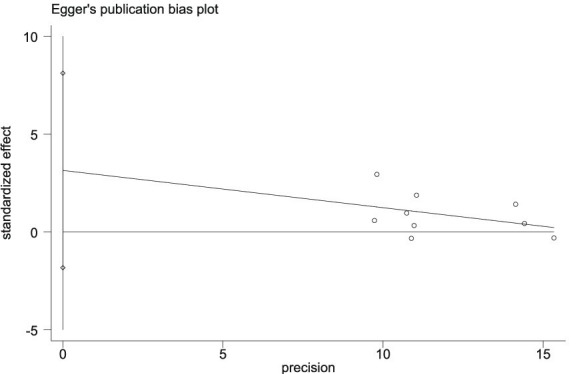
Egger’s test results of walking speed outcome indicators.

Due to the limited number of included studies, publication bias was assessed only for the walking speed outcome. Funnel plot and Egger’s test analyses indicated that the 9 scatter points were distributed on both sides of the centerline (Figure 18). Additionally, Egger’s test showed a result of *p* = 0.178 (Figure 19, Supplementary Figure 6), suggesting no substantial evidence of publication bias in the included studies.

## 4 Discussion

This systematic review and meta-analysis included a total of 13 randomized controlled trials, encompassing 380 patients with Parkinson’s disease, aiming to investigate the effects of wearable devices on gait, balance, motor function, and quality of life in this population. The results of this study suggest that wearable device interventions may selectively improve certain gait and motor outcomes in PwPD. Specifically, wearable device training was associated with a significant increase in walking speed, a notable reduction in TUG duration, and a significant improvement in UPDRS-III scores. However, the results for stride length, step length, step cadence, FOGQ scores, BBS, Mini-BESTest, DS time, 6MWT and PDQ did not reach statistical significance, suggesting that the therapeutic benefits of wearable devices may vary depending on the specific outcome measures, intervention protocols, and device characteristics employed.

### 4.1 Methodological quality and considerations

Assessment of the 13 included studies using the Cochrane Risk of Bias Tool revealed methodological limitations that should inform future trial design. Allocation concealment was inadequately reported in four studies. Future trials should implement centralized web-based or telephone randomization, or sequentially numbered opaque sealed envelopes, to prevent selection bias and avoid overestimation of treatment effects.

Blinding poses inherent challenges in wearable device research due to the visible nature of devices. Future studies should prioritize assessor blinding for subjective outcomes such as UPDRS-III and PDQ, as these are particularly susceptible to detection bias. When participant blinding is unfeasible, incorporating objective outcomes—such as instrumented gait parameters derived from inertial sensors—can effectively mitigate bias risk. Additionally, future trials should register protocols prospectively, adhere to CONSORT reporting guidelines, and explicitly document funding sources, sample size calculations, and any protocol deviations to enhance transparency, reproducibility, and overall methodological rigor.

### 4.2 The impact of wearable device on gait capacity in PwPD

This meta-analysis evaluated the effects of wearable devices on gait capacity in patients with Parkinson’s disease using multiple outcome measures, including walking speed, stride length, step length, step cadence, and FOGQ. Walking speed appeared to be significantly improved following wearable device interventions. This finding seems to be consistent with previous meta-analyses demonstrating that wearable device-based walking programs significantly improve gait speed in older adults, with particularly pronounced benefits in PwPD (42). The improvement in walking speed may be attributed to the compensatory mechanisms offered by wearable devices. By providing real-time sensory feedback—such as auditory, visual, or proprioceptive cues—these devices help bypass impaired basal ganglia function and facilitate movement initiation (43). Rhythmic auditory stimulation, in particular, has been shown to enhance gait velocity by promoting more regular step timing and reducing stride-to-stride variability, with responsive patients demonstrating cerebellar and angular gyrus activation without basal ganglia network connectivity loss (44, 45).

However, stride length, step length, step cadence, and FOGQ scores did not reach statistical significance. These findings are consistent with those reported by Zhang et al. (22), whose meta-analysis of wearable cueing devices in PwPD failed to detect significant effects on stride length. The potential reason for this consistency may be that both meta-analyses included a broad range of device types with varying feedback modalities, whereas analyses focused on specific sensor types have shown more pronounced effects on stride parameters. Different device types may exert distinct effects on gait subdomains; for instance, vibration stimulation has been shown to reduce left–right asymmetry but its effects on stride length vary among individuals. Additionally, the lack of significant improvement in FOGQ may reflect the multifactorial nature of freezing episodes. Research indicates that freezing of gait is linked to anomalies in pedunculopontine nucleus functional connectivity and involves cognitive and motor components that may not be fully addressed by sensory feedback alone (46). Wearable devices primarily target motor execution deficits but may have limited influence on the attentional dysfunction that often precipitates freezing (47). Future studies should consider multimodal interventions combining wearable technology with cognitive training to address the complex pathophysiology underlying gait impairment in PwPD.

### 4.3 The effects of wearable device on balance ability in PwPD

This meta-analysis evaluated the effects of wearable devices on balance ability in patients with Parkinson’s disease using the BBS, Mini-BESTest, TUGT and DS time. The key findings are as follows: The TUGT appeared to show significant improvement following wearable device interventions. This finding seems to be consistent with previous studies demonstrating that wearable device-based interventions may effectively enhance dynamic balance control in PwPD (48). The observed improvement in TUGT might be attributed to the real-time sensory feedback mechanisms provided by wearable devices. By delivering rhythmic auditory, visual, or vibrotactile cues, these devices may compensate for impaired internal cueing mechanisms in the basal ganglia, facilitating more automatic movement initiation and execution during complex tasks such as standing up, walking, turning, and sitting down (23).

However, BBS, Mini-BESTest, and DS time did not reach statistical significance. These findings are consistent with Wu et al. (49), whose research demonstrated that a borderline improvement in balance favoring wearable devices, but the confidence and prediction intervals were wide and included no effect, indicating substantial uncertainty. The potential reason may be that, among the included trials, little study explicitly designed the wearable intervention as a balance-focused training program, whereas the others primarily targeted gait initiation or step regulation; thus, the cueing content and training priorities may not have been optimal for producing measurable changes on balance scales. What’smore, the lack of significant improvement in BBS may reflect that wearable cueing devices primarily target dynamic balance control rather than the static balance components assessed by the BBS (50). Mechanistically, balance control in PwPD is compromised by impaired proprioceptive processing and delayed postural responses. Wearable devices that provide continuous sensory feedback may help compensate for these deficits by enhancing sensorimotor integration and facilitating more rapid postural adjustments (49). Nevertheless, the heterogeneity in device types and training protocols across studies likely contributes to the variability in observed balance outcomes. Future studies should standardize intervention protocols and employ comprehensive balance assessment batteries to better elucidate the differential effects of wearable devices on static and dynamic balance components in PwPD.

### 4.4 The effects of wearable device on motor function in PwPD

This meta-analysis evaluated the effects of wearable devices on motor function in patients with Parkinson’s disease using the UPDRS-III and the 6MWT. The key findings are as follows: UPDRS-III scores appeared to show significant improvement following wearable device interventions. The improvement in UPDRS-III may be attributed to the neuroplastic effects induced by repetitive, task-specific training enabled by wearable devices (51). By providing real-time sensory feedback, these devices facilitate closed-loop motor learning, which promotes corticostriatal plasticity and enhances neural circuit efficiency (52). Furthermore, wearable devices that deliver rhythmic cueing have been shown to reduce bradykinesia by compensating for impaired internal timing mechanisms in the basal ganglia, thereby improving limb coordination and axial motor control (50).

However, when we further analyzed different scales, this effect did not reach statistical significance when the more contemporary MDS-UPDRS-III was used. Several factors might help explain this observed discrepancy. First, the MDS-UPDRS-III includes additional items and more detailed scoring anchors, making it potentially a more stringent instrument that may require larger effect sizes or sample sizes to detect significant changes. Second, the smaller number of studies using the MDS-UPDRS-III might have limited statistical power. Third, differences in intervention protocols and device characteristics across studies may have differentially influenced scores on the two scales. Taken together, while wearable devices may improve motor function, the evidence appears to be less robust when assessed with the more rigorous MDS-UPDRS-III, suggesting that further high-quality studies are warranted.

However, 6MWT results did not reach statistical significance. The lack of significant improvement in 6MWT may reflect that this outcome measure assesses endurance capacity and overall functional mobility, which may require longer intervention periods to achieve detectable changes compared to the more targeted motor assessments captured by UPDRS-III. Additionally, the 6MWT is influenced by multiple factors including cardiorespiratory fitness, motivation, and fatigue, which may not be directly targeted by wearable device interventions (53). Mechanistically, while wearable devices can improve specific motor subdomains such as gait initiation and rhythmicity, translating these improvements into sustained endurance gains likely requires more prolonged and intensive training protocols (29). Future studies should consider extending intervention durations and incorporating aerobic components to better address endurance capacity in PwPD.

### 4.5 The effects of wearable device on quality of life in PwPD

Across the included trials, wearable device interventions did not result in statistically significant improvements in PDQ scores. These findings is consistent with Wu et al. (49). This pattern suggests that short-term, device-focused interventions may not be sufficient to translate modest improvements in gait or motor scores into perceived gains in overall quality of life. The lack of significant improvement may be attributed to several factors. First, nonmotor symptoms such as anxiety, depression, and cognitive impairment—which substantially influence quality of life—were rarely targeted explicitly in the included trials. Second, psychosocial and environmental factors, including social support, community participation, and home environment adaptations, were not addressed by wearable interventions alone. Third, the relatively short intervention durations may have been inadequate to achieve meaningful changes in patient-reported outcomes, which often require sustained behavioral and psychological adaptations.

From a clinical perspective, these findings underscore that wearable devices are unlikely to replace comprehensive multidisciplinary rehabilitation. Rather, they may serve as adjunct tools within a broader program that also addresses cognition, mood, balance confidence, and environmental adaptation (54). Future studies should integrate patient-reported outcomes as primary endpoints, extend intervention and follow-up periods, and combine wearable technology with psychosocial support and environmental modifications to optimize meaningful improvements in quality of life for PwPD.

### 4.6 The optimal dose of wearable device for PwPD

#### 4.6.1 Duration of continuous intervention

This study indicates that the effects of wearable devices on walking speed in patients with Parkinson’s disease vary significantly depending on the duration of continuous intervention. Specifically, interventions lasting 7–12 weeks demonstrated a significant improvement in walking speed, whereas short-term interventions (1–6 weeks) did not achieve statistically significant training effects. This finding is consistent with the results reported by Yuan et al. (55). The lack of effect in short-term interventions may be because 1–6 weeks are insufficient to induce the neuroplastic changes necessary for sustained gait improvement, as motor learning and corticostriatal plasticity require repetitive, task-specific practice over an extended period. Conversely, long term intervention may be associated with decreased adherence due to technical challenges, loss of motivation, or increased burden of daily use (56). Notably, both subgroups exhibited low heterogeneity, suggesting consistent effects across studies within each duration category. Overall, a wearable device intervention lasting 7–12 weeks may represent a better time window for enhancing gait capacity in PwPD. However, further high-quality randomized controlled trials with standardized protocols are needed to confirm these findings and to explore the long-term sustainability of training effects beyond 12 weeks.

#### 4.6.2 Weekly intervention frequency

The World Health Organization recommends that older adults engage in at least three sessions of moderate-intensity physical activity per week to maintain physical function and reduce fall risk (57). Similarly, wearable device-based training, which combines physical exercise with real-time sensory feedback, represents a form of technology-assisted physical activity that may follow comparable dose–response principles. This finding is broadly consistent with the results reported by Li et al. (58). Multiple randomized controlled trials have demonstrated that wearable device interventions performed at least three times per week may significantly improve gait and motor function in Parkinson’s disease (59–61). These conclusions were partially supported in our study, which explored the intervention effects of different training frequencies. Our findings indicate that wearable device interventions performed 3–5 times per week resulted in a modest but statistically significant improvement in walking speed, whereas frequencies of 1–2 or 6–7 times per week did not. However, the lower 95% CI bound was zero, the point estimate was near the commonly reported minimal clinically important difference for gait speed in PD, and the subgroup included only five studies with small samples. Therefore, these results should be interpreted with caution. The lack of effect at lower frequencies may reflect insufficient motor learning, while higher frequencies might increase fatigue or reduce adherence. Notably, the three subgroups exhibited low to moderate heterogeneity, suggesting consistent effects across studies within each frequency category. Taken together, wearable device interventions performed 3–5 times per week may represent a potentially favorable training frequency for enhancing gait capacity in PwPD, although this conclusion requires confirmation in larger, adequately powered studies. Future studies should systematically investigate the interaction between training frequency and intervention duration, while also considering individual patient characteristics such as disease severity and baseline functional status to optimize personalized rehabilitation protocols.

#### 4.6.3 Technological heterogeneity and potential mechanisms of wearable devices

Although wearable devices differ substantially in their mechanisms of action, neither device category nor feedback modality significantly modified the intervention effect on walking speed in the present subgroup analyses. Active sensor-based feedback devices may improve gait by providing real-time auditory or visual cues that help patients adjust step length, cadence, rhythm, or postural control during walking. Passive proprioceptive devices, such as textured insoles, proprioceptive stimulation devices, or sensory-motor orthotics, are more likely to act through enhanced plantar sensory input, proprioceptive afference, and sensorimotor integration. In contrast, exoskeleton wearable devices may provide mechanical assistance, movement guidance, and repetitive task-specific gait practice. Therefore, the term “wearable devices” should not be interpreted as representing a single intervention mechanism, but rather as an umbrella category covering technologies with different therapeutic targets.

The absence of statistically significant subgroup differences should be interpreted cautiously. Several subgroups included only one or two studies, resulting in limited statistical power and wide confidence intervals. Therefore, the lack of significant subgroup effects does not necessarily indicate equivalence between device types or feedback modalities. Rather, it suggests that the available evidence is insufficient to determine whether one specific category of wearable device or feedback strategy is superior to another.

These findings partly explain why the pooled effects differed across outcomes. Wearable technologies may produce more immediate effects on walking speed through external cueing, sensory augmentation, or mechanical assistance, whereas outcomes such as balance scales, freezing of gait, endurance, or quality of life may require longer training exposure, more targeted intervention content, or comprehensive multidisciplinary rehabilitation. Future trials should directly compare different device categories and feedback modalities using standardized outcomes and adequately powered designs.

## 5 Limitations

Several limitations of this systematic review and meta-analysis should be acknowledged. First, the number of eligible randomized controlled trials and total sample size for each outcome were modest, limiting statistical power and precision, particularly for balance and quality-of-life measures, and precluding formal publication bias assessments for most outcomes. Second, substantial clinical and methodological heterogeneity existed across studies in terms of device types, feedback modalities, and intervention protocols, which may have diluted device-specific effects and limits generalizability. Third, intervention periods and follow-up durations were relatively short, leaving the long-term sustainability of observed benefits unknown. Fourth, the inherent difficulty of blinding participants and personnel in wearable device trials may have introduced performance and detection biases for subjective outcomes. Fifth, three crossover trials were excluded from the primary meta-analysis because the published reports did not provide sufficient paired data to reconstruct within-participant treatment effects. These studies were therefore summarized in the Supplementary materials. Although their findings were generally consistent with the direction of the main analysis, they should be interpreted cautiously because they could not be incorporated using the recommended paired crossover approach.

## 6 Conclusion

This systematic review and meta-analysis of 13 randomized controlled trials encompassing 380 patients with Parkinson’s disease evaluated the effects of wearable device interventions on gait, balance, motor function, and quality of life. The findings suggest that wearable devices may improve walking speed, TUGT performance, and UPDRS-III scores, indicating potential benefits for select gait parameters, dynamic balance, and overall motor function. However, no significant improvements were observed for stride length, step length, step cadence, FOGQ, BBS, Mini-BESTest, DS time, 6MWT, or PDQ scores. Subgroup analyses suggested that interventions lasting 6–12 weeks and those performed 3–5 times per week may represent the optimal dose for enhancing walking speed, although this finding requires confirmation in future studies.

From a clinical perspective, wearable devices may serve as effective adjunctive tools within comprehensive multidisciplinary rehabilitation programs, particularly for improving walking capacity and motor function. However, the evidence base remains limited, primarily due to small sample sizes, methodological limitations, and heterogeneity in device types and intervention protocols. Future large-scale, high-quality randomized controlled trials with standardized protocols, extended follow-up periods are warranted to confirm these findings, establish long-term sustainability, and optimize the implementation of wearable technologies in routine Parkinson’s disease care.

## Data Availability

The original contributions presented in the study are included in the article/Supplementary material, further inquiries can be directed to the corresponding author.
