# Supplementary material for: Effects of wearable devices on gait, balance, and motor function in people with Parkinson’s disease: a systematic review and meta-analysis
Source: Front Public Health. 2026 Jul 15;14:1846005. doi: 10.3389/fpubh.2026.1846005 (PMC13415929; doi:10.3389/fpubh.2026.1846005)
Supplement: Supplementary file 1 [file Supplementary_file_1.DOCX]

**Supplementary material 1：**

**1. Pubmed**

#1 ("Parkinson Disease"[Mesh]) OR (((((((((((Idiopathic Parkinson Disease[Title/Abstract]) OR (Idiopathic Parkinson's Disease[Title/Abstract])) OR (Lewy Body Parkinson Disease[Title/Abstract])) OR (Lewy Body Parkinson's Disease[Title/Abstract])) OR (Paralysis Agitans[Title/Abstract])) OR (Parkinson Disease, Idiopathic[Title/Abstract])) OR (Parkinson's Disease[Title/Abstract])) OR (Parkinson's Disease, Idiopathic[Title/Abstract])) OR (Parkinson's Disease, Lewy Body[Title/Abstract])) OR (Primary Parkinsonism[Title/Abstract])) OR (Parkinsonism, Primary[Title/Abstract])) ([149,154](https://pubmed.ncbi.nlm.nih.gov/?term=("Parkinson+Disease"[Mesh])+OR+(((((((((((Idiopathic+Parkinson+Disease[Title/Abstract])+OR+(Idiopathic+Parkinson's+Disease[Title/Abstract]))+OR+(Lewy+Body+Parkinson+Disease[Title/Abstract]))+OR+(Lewy+Body+Parkinson's+Disease[Title/Abstract]))+OR+(Paralysis+Agitans[Title/Abstract]))+OR+(Parkinson+Disease,+Idiopathic[Title/Abstract]))+OR+(Parkinson's+Disease[Title/Abstract]))+OR+(Parkinson's+Disease,+Idiopathic[Title/Abstract]))+OR+(Parkinson's+Disease,+Lewy+Body[Title/Abstract]))+OR+(Primary+Parkinsonism[Title/Abstract]))+OR+(Parkinsonism,+Primary[Title/Abstract]))&sort=))

#2 ((((((((("Wearable Electronic Devices"[Mesh]) OR ((((((((((((((Device, Wearable Electronic[Title/Abstract]) OR (Electronic Device, Wearable[Title/Abstract])) OR (Wearable Electronic Device[Title/Abstract])) OR (Wearable Devices[Title/Abstract])) OR (Device, Wearable[Title/Abstract])) OR (Wearable Device[Title/Abstract])) OR (Wearable Technology[Title/Abstract])) OR (Technology, Wearable[Title/Abstract])) OR (Wearable Technologies[Title/Abstract])) OR (Electronic Skin[Title/Abstract])) OR (Skin, Electronic[Title/Abstract])) OR (Wearable Computer[Title/Abstract])) OR (Computer, Wearable[Title/Abstract])) OR (Wearable Computers[Title/Abstract]))) OR (Motion Capture Devices[Title/Abstract])) OR (Inertial Measurement Unit[Title/Abstract])) OR (Acceleromet*[Title/Abstract])) OR (gyroscope*[Title/Abstract])) OR (sensor*[Title/Abstract])) OR (shoe*[Title/Abstract])) OR (Insole*[Title/Abstract])) OR (activity tracker*[Title/Abstract]) (620,275)

#3 (((("Clinical Trial" [Publication Type]) OR "Clinical Trials as Topic"[Mesh]) OR "Random Allocation"[Mesh]) OR "Randomized Controlled Trial" [Publication Type]) OR ((((random*[Title/Abstract]) OR (Clinical Trial[Title/Abstract])) OR (placebo[Title/Abstract])) OR (RCT[Title/Abstract])) ([2,568,095)](https://pubmed.ncbi.nlm.nih.gov/?term=(((("Clinical+Trial"+[Publication+Type])+OR+"Clinical+Trials+as+Topic"[Mesh])+OR+"Random+Allocation"[Mesh])+OR+"Randomized+Controlled+Trial"+[Publication+Type])+OR+(((random*[Title/Abstract])+OR+(Clinical+Trial[Title/Abstract]))+OR+(placebo[Title/Abstract]))&sort=)

#4 #1 AND #2 AND #3 (702)

**2.Web of Science**

#1 TS=(Parkinson Disease) OR TS=(parkinson*) OR TS=(Paralysis Agitans) (291,587)

#2 TS=(Wearable*) OR TS=(Electronic Skin) OR TS=(Motion Capture Devices) OR TS=(Inertial Measurement Unit) OR TS=(Acceleromet*) OR TS=(gyroscope*) OR TS=(sensor*) OR TS=(shoe*) OR TS=(Insole*) OR TS=(activity tracker*) (2,548,739)

#3 TS=(clinical trial*) OR TS=(random*) OR TS=(placebo*) OR TS=(RCT) (4,211,114)

#4 #1 AND #2 AND #3 (1856)

**3.Cochrane library**

#1 MeSH descriptor: [Parkinson Disease] in all MeSH products OR (Parkinson Disease):ti,ab,kw OR (parkinson*):ti,ab,kw OR (Paralysis Agitans):ti,ab,kw (15,554)

#2 MeSH descriptor: [Wearable Electronic Devices] in all MeSH products OR (Wearable*):ti,ab,kw OR (Electronic Skin):ti,ab,kw OR (Motion Capture Devices):ti,ab,kw OR (Inertial Measurement Unit):ti,ab,kw OR (Acceleromet*):ti,ab,kw OR (gyroscope*):ti,ab,kw OR (sensor*):ti,ab,kw OR (shoe*):ti,ab,kw OR (Insole*):ti,ab,kw OR (activity tracker*):ti,ab,kw (51,398)

#3 (clinical trial*):ti,ab,kw OR (random*):ti,ab,kw OR (placebo*):ti,ab,kw OR (RCT):ti,ab,kw (1,651,883)

#4 #1 AND #2 AND #3 (806)

**4. Embase**

#1 'parkinson disease':ab,ti OR parkinson*:ab,ti OR 'paralysis agitans':ab,ti (246,248)

#2 wearable*:ab,ti OR 'electronic skin':ab,ti OR 'motion capture devices':ab,ti OR 'inertial measurement unit':ab,ti OR acceleromet*:ab,ti OR gyroscope*:ab,ti OR sensor*:ab,ti OR shoe*:ab,ti OR insole*:ab,ti OR 'activity tracker*':ab,ti (721,610)

#3 'clinical trial*':ab,ti OR random*:ab,ti OR placebo*:ab,ti OR rct:ab,ti (3,254,211)

#4 #1 AND #2 AND #3 (1509)

**5. China National Knowledge Infrastructure (CNKI)**

#1 (主题：帕金森） (39,539)

#2 (主题:可穿戴)OR(主题:传感器)OR(主题:追踪器)OR(主题:加速度计) (702,041)

#3 #1 AND #2 (290)

**6. Wan Fang**

#1 [主题:(帕金森)](http://gfffhcbe257df071c4bc8spuwcou5pofuv6pbq.ffgi.hbpu.wttczd-86544418598.com/advanced-search/paper?q=%E4%B8%BB%E9%A2%98:(%E5%B8%95%E9%87%91%E6%A3%AE)&searchtype=expert&type=["periodical","thesis","conference"]&chineseEnglishExpand=true" \t "http://gfffhcbe257df071c4bc8spuwcou5pofuv6pbq.ffgi.hbpu.wttczd-86544418598.com/advanced-search/_blank) (51,037)

#2 [主题:(可穿戴) or 主题:(传感器) or 主题:(追踪器) or 主题:(加速度计)](http://gfffhcbe257df071c4bc8spuwcou5pofuv6pbq.ffgi.hbpu.wttczd-86544418598.com/advanced-search/paper?q=%E4%B8%BB%E9%A2%98:(%E5%8F%AF%E7%A9%BF%E6%88%B4) or %E4%B8%BB%E9%A2%98:(%E4%BC%A0%E6%84%9F%E5%99%A8) or %E4%B8%BB%E9%A2%98:(%E8%BF%BD%E8%B8%AA%E5%99%A8) or %E4%B8%BB%E9%A2%98:(%E5%8A%A0%E9%80%9F%E5%BA%A6%E8%AE%A1)&searchtype=expert&type=["periodical","thesis","conference"]&chineseEnglishExpand=true" \t "http://gfffhcbe257df071c4bc8spuwcou5pofuv6pbq.ffgi.hbpu.wttczd-86544418598.com/advanced-search/_blank) (1,000,489)

#3 #1 AND #2 (448)

**7. VIP**

#1 (常用字段（标/关/摘/主题词）:帕金森) (69,701)

#2 (常用字段（标/关/摘/主题词）:可穿戴 OR 常用字段（标/关/摘/主题词）:传感器 OR 常用字段（标/关/摘/主题词）:追踪器 OR 常用字段（标/关/摘/主题词）:加速度计)  (150,808)

#3 #1 AND #2 (363)

**8. SinoMed**

#1 "帕金森"[全部字段:智能] (46,803)

#2 "可穿戴"[全部字段:智能] OR "传感器"[全部字段:智能] OR "追踪器"[全部字段:智能] OR "加速度计"[全部字段:智能] (21,728)

#3 #1 AND #2 (95)
