# Supplementary material for: Effects of wearable devices on gait, balance, and motor function in people with Parkinson’s disease: a systematic review and meta-analysis
Source: Front Public Health. 2026 Jul 15;14:1846005. doi: 10.3389/fpubh.2026.1846005 (PMC13415929; doi:10.3389/fpubh.2026.1846005)
Supplement: Supplementary file 2 [file Supplementary_file_2.DOCX]

**Supplementary material 2：**


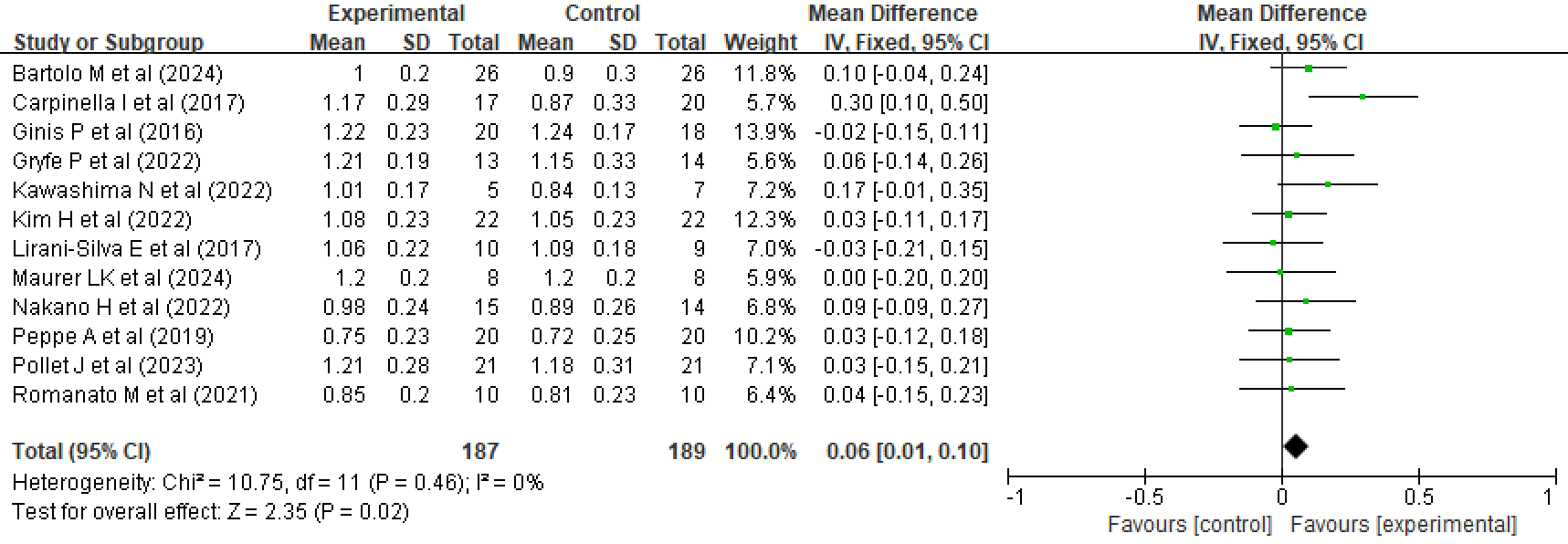


Supplementary Figure 1: Forest plots for walking speed (Add 3 crossover trials).


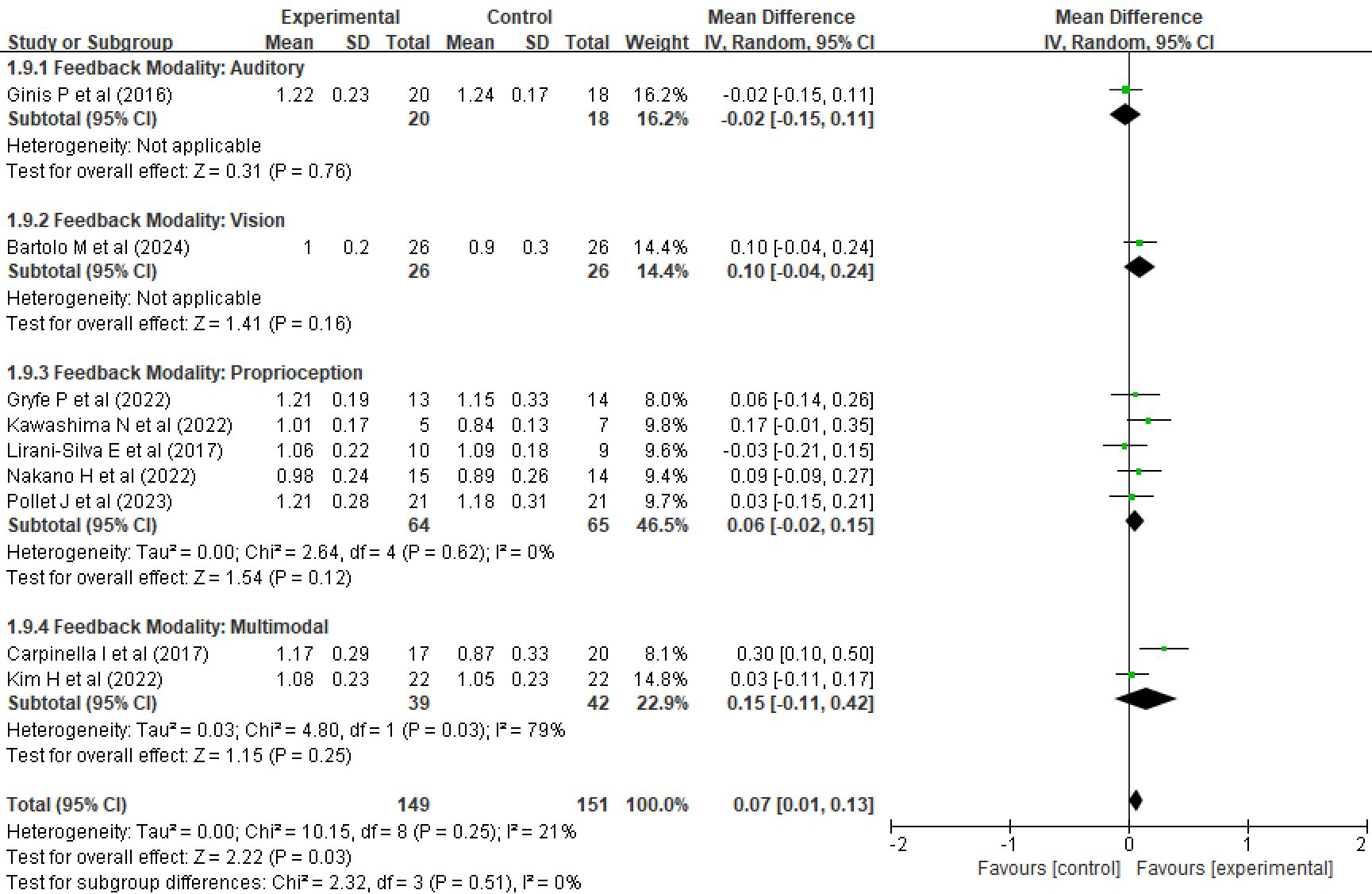


Supplementary Figure 2: Subgroup analysis of the effect of wearable device on the walking speed in PwPD based on feedback modality.


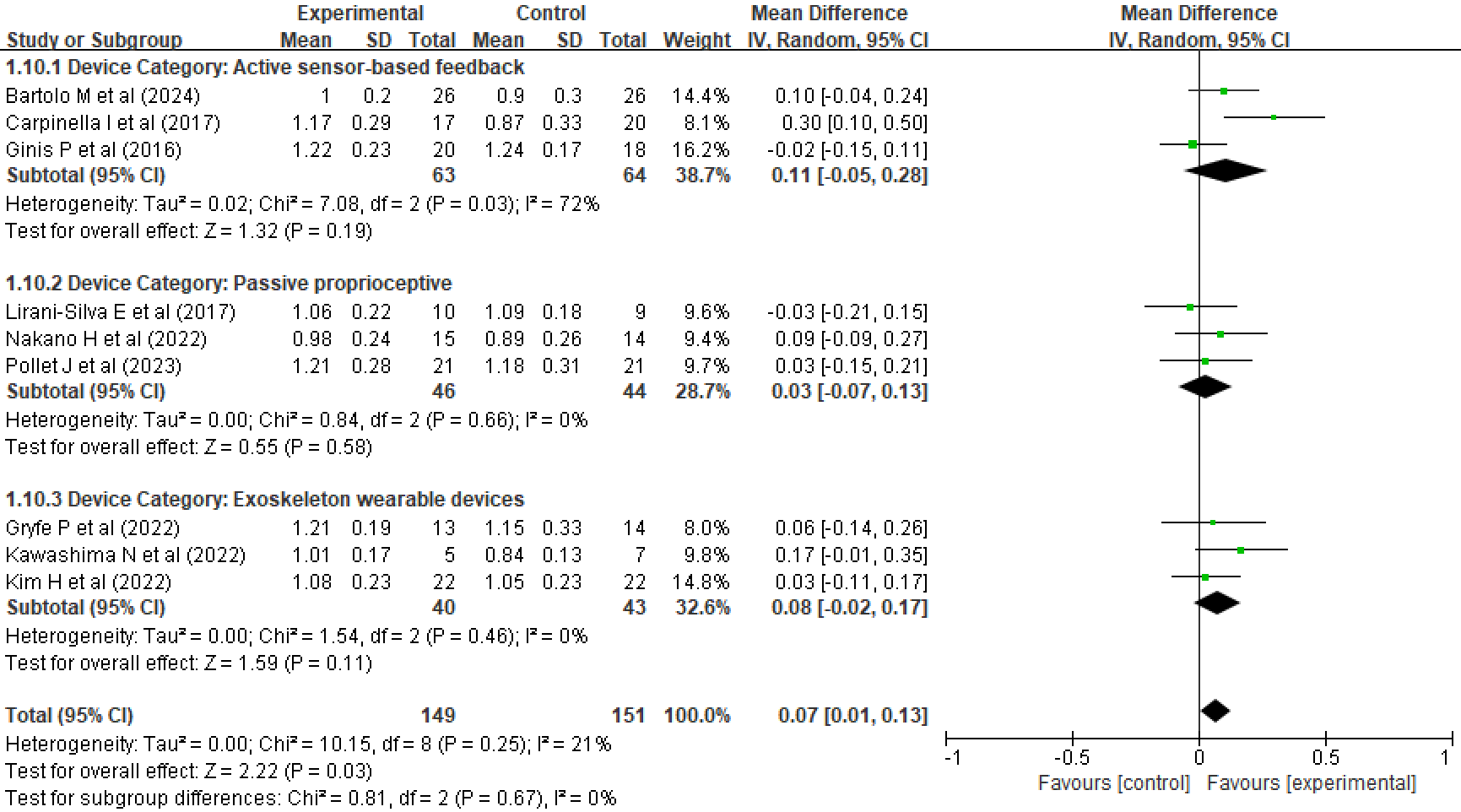


Supplementary Figure 3: Subgroup analysis of the effect of wearable device on the walking speed in PwPD based on device category.


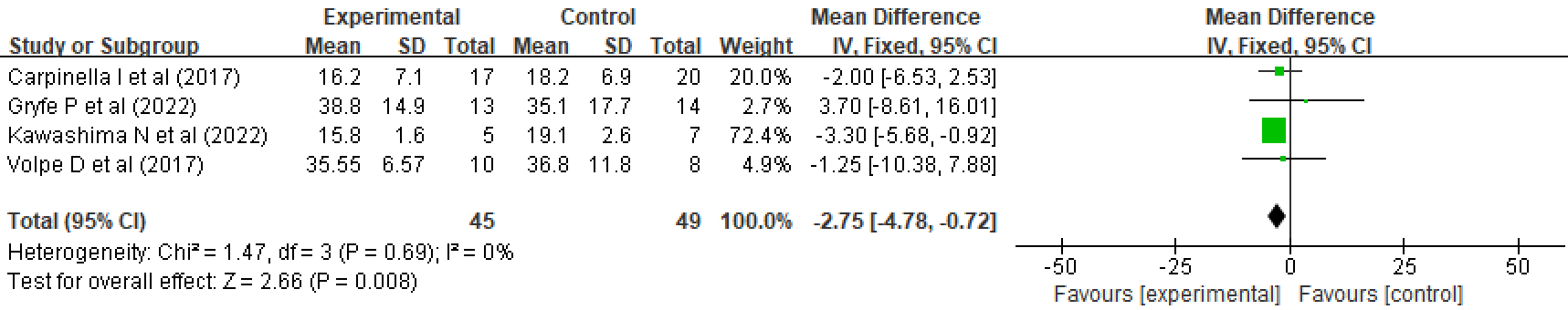


Supplementary Figure 4: Forest plots for UPDRS Ⅲ.


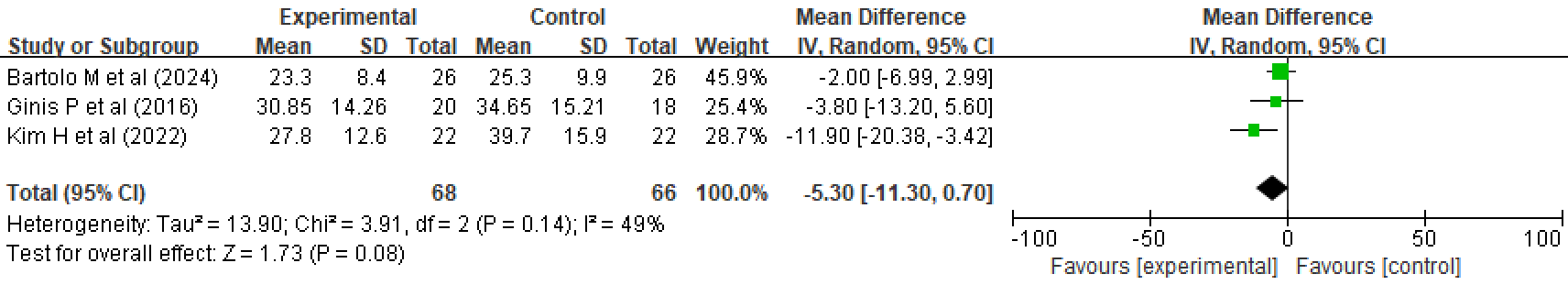


Supplementary Figure 5: Forest plots for MDS-UPDRS Ⅲ.


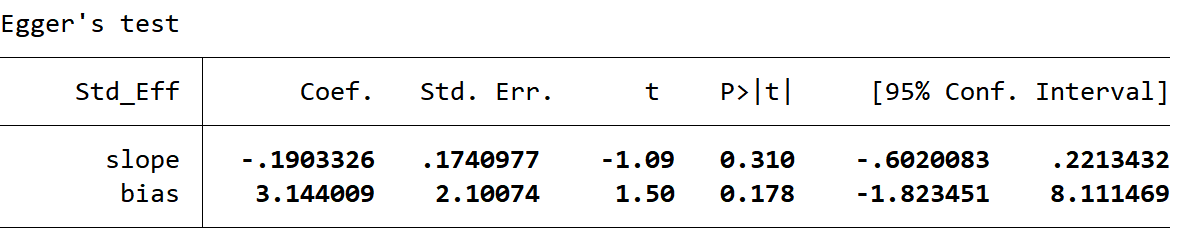
Supplementary Figure 6：Egger's test results of walking speed outcome indicators
